# Preliminary Study on Lutetium-177 and Gold Nanoparticles: Apoptosis and Radiation Enhancement in Hepatic Cancer Cell Line

**DOI:** 10.3390/cimb46110727

**Published:** 2024-10-30

**Authors:** Maria Anthi Kouri, Anastasios Georgopoulos, George E. Manios, Eirini Maratou, Aris Spathis, Sofia Chatziioannou, Kalliopi Platoni, Efstathios P. Efstathopoulos

**Affiliations:** 1 2nd Department of Radiology, Medical Physics Unit, Medical School, National and Kapodistrian University of Athens, 1 Rimini Str., 12462 Athens, Greece; mariakouri90@gmail.com (M.A.K.); anastgeorgopoulos@gmail.com (A.G.); ge.manios@gmail.com (G.E.M.); sofiac@med.uoa.gr (S.C.); kplatoni@med.uoa.gr (K.P.); 2 Medical Physics, General Hospital GHA Korgialeneio Mpenakeio-Hellenic Red Cross, Athanasaki 11, 11526 Athens, Greece; 3 Clinical Biochemistry Laboratory, Attikon General Hospital, Medical School, National and Kapodistrian University of Athens, 12462 Athens, Greece; maratou@hotmail.com; 4 2nd Department of Pathology, School of Medicine, Attikon University Hospital, National and Kapoditrian University of Athens, 12462 Athens, Greece; arisspa@gmail.com; 5 Department of Radiology, Nuclear Medicine Section, Baylor College of Medicine, St. Luke’s Episcopal Hospital, Houston, TX 77030, USA

**Keywords:** gold nanoparticles, Lutetium-177, radionuclide therapy, molecular therapy, HepG2 cancer cell line, gold nanospheres, radiosensitization, apoptosis, radiation enhancement ratio (RER)

## Abstract

This study investigates a novel approach toward enhancing the efficacy of Lutetium-177 (Lu-177) radiopharmaceutical therapy by combining it with gold nanoparticles (AuNPs) in the HepG2 hepatic cancer cell line. Lu-177, known for its effective β radiation, also emits gamma rays at energies (113 keV and 208 keV) near the photoelectric absorption range, suggesting potential for targeted and localized radiation enhancement when used in conjunction with AuNPs. Thus, HepG2 cells were treated at two different activity levels (74 MBq and 148 MBq), with Lu-177 alone, with a combination of Lu-177 and AuNPs in two sizes (10 nm and 50 nm), while some received no treatment. Treatment efficacy was assessed by quantifying the radiation enhancement ratio (RER) and the apoptosis levels. The results reveal that combining Lu-177 with AuNPs significantly increases cell death and apoptosis compared to Lu-177 alone, with 10 nm AuNPs demonstrating superior effectiveness. Additionally, varying Lu-177 activity levels influenced the treatment outcomes, with higher activity levels further augmenting the therapeutic impact of combined therapy. These findings underscore the potential of utilizing Lu-177’s beta, but also gamma, emissions, traditionally considered non-therapeutic, for localized radiation enhancement when combined with AuNPs. This novel strategy leverages Lu-177 as an internal irradiator to exploit gamma radiation for a targeted therapeutic advantage without requiring nanoparticle functionalization. The study provides a promising approach to improving radionuclide therapy and sets the stage for future research aimed at optimizing cancer treatments through the combined use of Lu-177 and AuNPs.

## 1. Introduction

Neuroendocrine tumors (NETs) comprise a heterogeneous group of malignancies that originate from neuroendocrine cells and are most commonly found in the gastrointestinal tract, pancreas, and lungs [1,2]. The liver is a frequent site of metastasis for NETs, particularly those arising from the gastrointestinal tract and pancreas, known as gastroenteropancreatic NETs (GEP-NETs) [3,4]. This predilection for liver metastasis is primarily due to the substantial blood supply the liver receives from the portal vein, which drains the gastrointestinal organs and serves as a conduit for disseminating tumor cells [5]. Hepatocellular carcinoma (HCC) is a major type of liver cancer that originates from hepatocytes and is characterized by a high incidence rate and poor prognosis due to its aggressive nature and limited treatment options [6,7,8]. Traditional therapies for these malignancies, including surgery, chemotherapy, and external beam radiation, often provide limited efficacy, particularly in advanced stages [9,10]. Recently, nuclear medicine has advanced significantly in the treatment of NETs and thus metastatic hepatic cancer, utilizing radiopharmaceuticals like Lutetium-177 (Lu-177) to deliver targeted radiation therapy at a molecular level [11,12,13].

Lu-177 is a radionuclide with favorable physical properties for targeted radionuclide therapy [14]. It decays by beta (β) emission to stable hafnium-177, with a half-life of 6.647 days, providing a balance between prolonged irradiation and manageable patient radiation safety [15,16]. The primary β emission has a maximum energy of 0.498 MeV, which is effective for damaging tumor cells within a relatively short range. Lu-177 also emits gamma (γ) radiation at 113 keV (6.2%) and 208 keV (11%), which can be potentially utilized for imaging and dosimetry rather than for therapeutic purposes [17,18].

The therapeutic effectiveness of Lu-177 in nuclear medicine is due to its β particles [19]. These particles have sufficient energy to cause ionization, resulting in direct DNA damage in cancer cells through single- and double-strand breaks [20,21]. Beta particles, unlike gamma rays, provide localized irradiation, which is crucial when delivering targeted therapy to tumors [22]. Gamma radiation, with its high penetrative ability, irradiates a broader area, increasing the risk of damage to surrounding healthy tissues [23]. Thus, β emissions are the preferred choice for radiopharmaceutical therapy, ensuring that radiation is deposited selectively within the tumor.

However, the gamma radiation emitted by Lu-177, which is not useful for therapeutic purposes, presents an interesting opportunity due to its emission energies. The gamma photons emitted by Lu-177 have energy levels close to the photoelectric absorption range, where the probability of photon interaction with high-atomic-number (Z) materials, such as gold, is significantly enhanced [24,25]. This characteristic makes gamma emissions a potential tool for inducing localized radiation enhancement within the tumor microenvironment.

Given these properties, in this study, we hypothesized that gold nanoparticles (AuNPs), which are known for their radioenhancement capabilities due to their high atomic number and favorable interactions with ionizing radiation, could be leveraged to exploit the therapeutic not-exploited gamma emissions from Lu-177 in addition to the therapeutically used beta particles. When irradiated at energies near the photoelectric threshold, gold nanoparticles (AuNPs) exhibit unique chemical, physical, and biological properties that lead to a cascade of secondary radiation effects [26]. This includes the emission of Auger electrons, which have an extremely short range in biological tissue (on the order of a few nanometers) and are highly effective in inducing multiple, irreparable double-strand and single-strand DNA breaks [27]. Concurrently, AuNPs promote the generation of reactive oxygen species (ROS), further contributing to indirect DNA damage and increasing oxidative stress within tumor cells [28]. Together, these mechanisms enhance cell death, particularly apoptosis, thereby potentially improving the therapeutic efficacy of the treatment [29,30].

This study aims to investigate the effects of an Lu-177 DOTATATE solution combined with AuNPs on hepatic cancer cell line HepG2, providing a complementary perspective to the existing research that mainly addresses the functionalization of AuNPs with Lutetium [31,32,33,34]. Instead of directly conjugating Lu-177 to the nanoparticles, our approach utilizes Lu-177 as an internal irradiator to harness its gamma and beta emissions for localized radioenhancement. By leveraging Lu-177 widely used beta emissions, but also its gamma emissions that are typically considered therapeutically redundant, we aim to enhance the radiation dose delivered specifically within the tumor microenvironment, thereby increasing cell death, with a particular emphasis on apoptosis. This novel strategy offers a unique method to improve the efficacy of radionuclide therapy by transforming otherwise “non-therapeutic” gamma emissions into a targeted therapeutic advantage, without the need for complex nanoparticle modifications.

## 2. Materials and Methods

### 2.1. Cell Culture

HepG2, a human hepatocellular carcinoma cell line, was obtained from the American Type Culture Collection (ATCC, Manassas, VA, USA). The cells were cultured in accordance with the protocol provided by the supplier at the Clinical Biochemistry Laboratory, Attikon General Hospital, Medical School, National and Kapodistrian University of Athens, Athens, Greece. The HepG2 cells were maintained in Dulbecco’s Modified Eagle Medium (DMEM), supplemented with 10% fetal bovine serum (FBS) and 1% penicillin–streptomycin. Cultures were incubated at 37 °C in a humidified atmosphere containing 5% CO_2_ until they reached approximately 80% confluency. Cells were subsequently harvested, counted, and seeded into 24-well plates at a density of 200,000 cells per well.

### 2.2. Gold Nanoparticles (AuNPs)

Two types of spherical gold nanoparticles, both sourced from NanoComposix (San Diego, CA, USA), were utilized. The first type consisted of 10 nm diameter nanospheres, and the second type comprised of 50 nm diameter nanospheres. Both types of AuNPs were functionalized with Methoxyl-PEG (mPEG) on their surface to enhance stability and biocompatibility [35]. Furthermore, mPEG coatings have proved to prevent clustering and agglomeration by creating a steric barrier, thereby reducing undesirable interactions between particles [36]. Additionally, PEGylation prolongs circulation time by minimizing protein adsorption and immune recognition, decreases immunogenicity, improves solubility in aqueous environments, and thus increases the overall possibility of AuNPs deposition in cell’s critical micro-organels [37]. According to the technical data provided by NanoComposix, both the 10 nm and 50 nm AuNPs are highly monodisperse, with polydispersity index (PDI) values below 0.1, indicating very good size uniformity. Additionally, DLS measurements indicate that the hydrodynamic diameters of the AuNPs fall within the range of 7–47 nm for both the 10 nm and the 50 nm particles, which aligns well with their nominal sizes. Moreover, the Z-potential measurements reveal that both types of AuNPs exhibit surface charges of ≤5 mV, confirming their stability in dispersion. For each experimental group involving gold nanoparticles, 5 μg/mL of the appropriate AuNPs was added to the wells. Cells were incubated with the AuNPs for 24 h to facilitate enhanced permeability and retention (EPR) within the cells.

### 2.3. Lutetium-177 Treatment

The Lu-177 DOTATATE radiopharmaceutical, was provided by the Nuclear Medicine and Molecular Imaging Department of Attikon University Hospital, Athens, Greece. For our experiments, we explored the effect of different activity levels on the process of radioenhancement and cell apoptosis by using two distinct activities: 74 MBq and 148 MBq. These activities were obtained by adjusting the quantities of the radiopharmaceutical solution used in the cell medium. The cell medium in the designated wells was infused with these specific concentrations of Lu-177. Following the infusion, the cells were incubated for 48 h with the assigned treatment of each group.

### 2.4. Experimental Design

**Figure 1:**
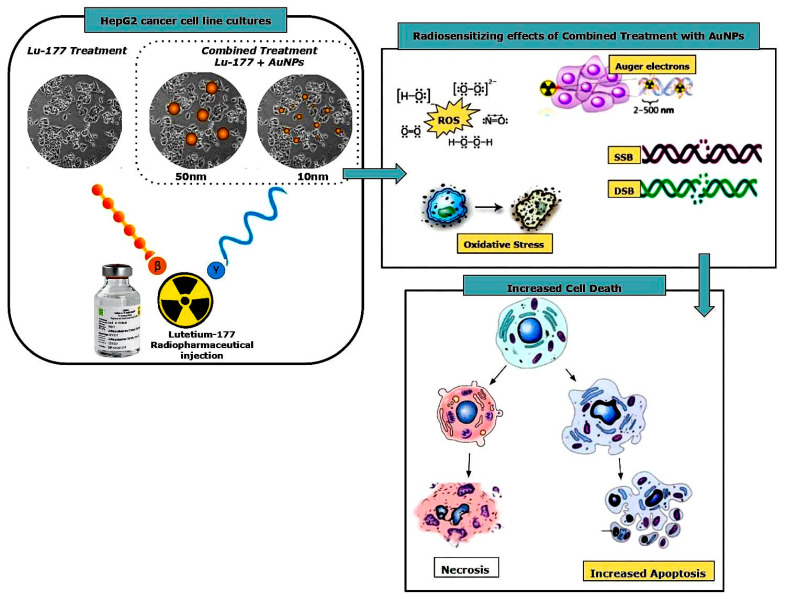
Experimental set up and radiosensitizing effects due to the use of combined treatment of Lu-177 and AuNPs of two distinct sizes: 50 nm and 10 nm.

Following the attainment of the desired confluency, the HepG2 cells in the 24-well plates were divided into 4 distinct experimental groups to assess the effects of Lu-177 and AuNPs on cellular viability and radiation biology effects. The first group, designated as the control, received no treatment. The second group was treated with Lu-177 alone. The third group received a combination of Lu-177 and 50 nm spherical AuNPs, while the fourth group was treated with Lu-177 in conjunction with 10 nm spherical AuNPs, as be depicted in Figure 1. For each experimental group, 1 set was treated with Lu-177 at an activity of 74 MBq, while a second set was treated with Lu-177 at an activity of 148 MBq.

### 2.5. Post-Treatment Analysis

Cell viability was analyzed using flow cytometry on an Omnicyt (Cytognos, Salamanca, Spain) at the 2nd Department of Pathology of the School of Medicine at Attikon University Hospital, in Athens, Greece. After 48 h of incubation with the different treatments, the cells were stained using the Annexin V assay (Alexa Fluor 488, Dead Cell Apoptosis Kit, Invitrogen, Thessaloniki, Greece) to evaluate and quantify cell death. This assay allowed for the differentiation and measurement of apoptosis and necrosis induced by ionizing radiation from Lu-177, applied at two different activity levels, both in the presence and absence of AuNPs of varying sizes.

### 2.6. Clonogenic Survival Assay and Data Processing

A clonogenic survival assay was conducted to evaluate the therapeutic effects of radiation on the survival of cancer cells with and without the presence of AuNPs, using different activities of the Lu-177 radiopharmaceutical. Unlike external radiation sources, such as a clinical LINAC, this study utilized Lu-177 as an internal irradiator for both the AuNPs and the cancer cells. The radiopharmaceutical served to deliver ionizing radiation internally, simulating the conditions of nuclear medicine treatments where Lu-177 functions as a targeted therapy. The relative cell surviving fraction was calculated with the aid of Equations (1) and (2):

(1) $$\mathrm{Platting Efficiency}\;(\mathrm{PE})(\%)\;=\;\frac{No.\;of\;Coloniesformed}{No.of\;cellsseeded}\;\times \;100$$

(2) $$\mathrm{Surviving Fraction}\;(\mathrm{SF})(\%)=\frac{No.\;of\;Colonies\;formed\;after\;treatment}{No.\;of\;cells\;seeded\;\times \;PE}\;\times \;100\;$$

Plating efficiency (PE) represents the ratio of the number of colonies formed to the number of cells initially seeded. Following treatment, the number of colonies counted is expressed in terms of PE, but redefined as the surviving fraction (SF).

To further assess the impact of Lu-177 treatment in terms of radiosensitization, with and without the presence of AuNPs, and to correlate these results with the physics of radiation interactions and radiation biology, the radiation enhancement ratio (RER) was utilized. Given that our experiment focused on radioactivity levels, as is commonly employed in the clinical practice of nuclear medicine, rather than on radiation doses, metrics such as the dose enhancement factor (DEF) or sensitization enhancement ratio (SER) were not applicable.

In this study, the RER was employed to characterize the radiobiological effects of AuNPs by comparing the survival fractions at different levels of activity, specifically 74 MBq and 148 MBq of Lu-177, with and without AuNPs. The radiation enhancement ratio (RER) is defined as the ratio of survival fractions in the absence of AuNPs to those with AuNPs, thereby directly illustrating the variation in biological response induced by the nanoparticles at specific levels of radioactivity. Although this parameter is limited to cell survival studies, it effectively portrays the influence of AuNPs on the radiobiological response at specific activity levels of Lu-177. The radiation enhancement ratio (RER) was calculated with the aid of Equation (3):

(3) $$\mathrm{RER}\;=\;\frac{SFconst}{SF\;Np}$$

### 2.7. Statistical Analysis

Statistical comparisons were performed for the survival fractions over activities, and for both the RER and apoptosis measurements under different experimental conditions (n = 4 biological replicates) using the non-parametric Wilcoxon–Mann–Whitney test. For the survival fraction analysis, statistical comparisons were made between the 2 Lu-177 activities (74 and 148 MBq) across all treatment groups (no treatment, Lu-177 treatment alone, and combined Lu-177 and AuNPs treatment with two different AuNP sizes). Additionally, statistical comparisons were performed among the different treatment groups. For the RER measurements, comparisons were made based on the Lu-177 activities and the sizes of the AuNPs used (10 nm, 50 nm). Differences were considered statistically significant at a *p*-value of less than 0.05. For the apoptosis measurements, we compared the results between the two Lu-177 activities injected (74, 148 MBq) and across the different treatments applied (no treatment, Lu-177 treatment, and combined Lu-177 and AuNPs treatment with two different AuNP sizes).

### 2.8. Radiation Safety and Waste Disposal

Upon the completion of the experimental procedures, all remnants of the Lu-177 radiopharmaceutical were meticulously extracted and disposed of in accordance with established radiation safety regulations. Strict precautions were implemented to ensure that no radioactive contamination occurred within the laboratory environment.

## 3. Results

### 3.1. Cell Survival

**Figure 2:**
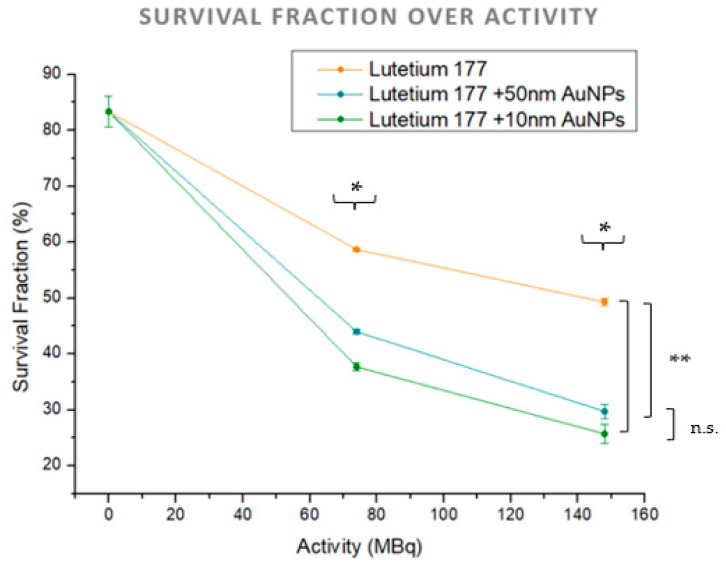
Survival fraction of HepG2 cancer cell line against the activity for Lu-177 treatment alone (orange), for the combined therapy of Lu-177 with 50 nm AuNPs (blue), and for the combined therapy of Lu-177 with 10 nm AuNPs (green). (*) Statistically significant difference (*p* < 0.05)—(comparison between different activities, 74 MBq vs. 148 MBq), (**) statistically significant difference (*p* < 0.05)—(comparison between treatments), and (n.s.) non-significant difference (*p* > 0.05).

Cell survival was evaluated by plotting the survival fraction of HepG2 cells against the activity of Lu-177 at 0 MBq (control), 74 MBq, and 148 MBq. Three distinct survival curves were generated within a single graph: one for Lu-177 treatment alone, another for the combined therapy of Lu-177 with 50 nm AuNPs, and the third for the combination of Lu-177 with 10 nm AuNPs, as depicted in Figure 2.

Statistical comparisons between the two Lu-177 activities (74 and 148 MBq) across all treatment groups (no treatment, Lu-177 treatment alone, and combined Lu-177 and AuNPs treatment with two different AuNP sizes) revealed a statistically significant difference (*p* = 0.03). Further analysis of the treatment groups showed a statistical significance between Lu-177 treatment alone and the combined treatment of Lu-177 with 50 nm AuNPs (*p* = 0.04), as well as between Lu-177 treatment alone and the combination with 10 nm AuNPs (*p* = 0.018). However, no statistical significance was found when comparing the combined treatments of Lu-177 with 50 nm and 10 nm AuNPs (*p* = 0.06).

The decision to plot the survival fraction over activity, rather than converting activities to doses using internal dosimetry, was made to align with the standard practices in nuclear medicine, where activity is the clinically relevant parameter. Consequently, we did not apply any radiobiological survival models, such as the linear–quadratic model, in this analysis. This approach allows for a direct interpretation of the effects of clinically used activities on cell survival.

### 3.2. Radiation Enhancement Ratio

**Figure 3:**
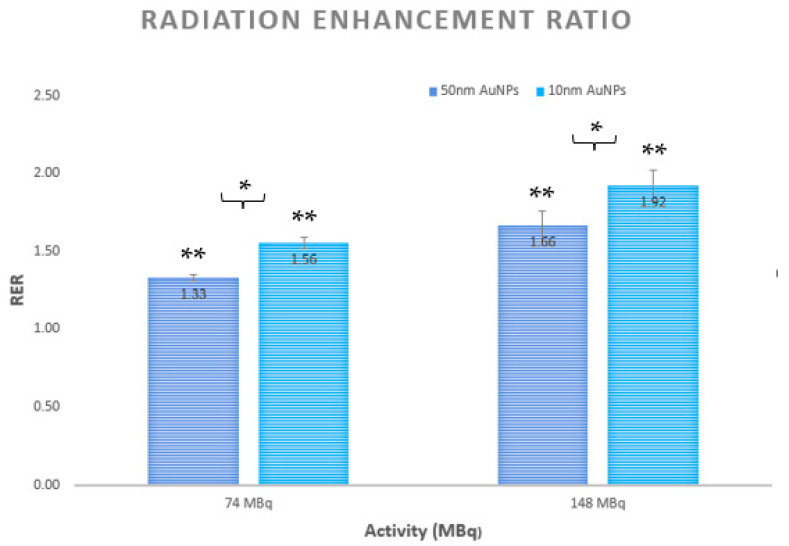
Radiation enhancement ratio (RER) over activity for the combined therapies of Lu-177 and AuNPs in the HepG2 cancer cell line. The RER is calculated and plotted for the sizes of 50 nm of AuNPs (purple) and for 10 nm of AuNPs (blue). (*) Statistically significant difference (*p* < 0.05)—(comparison between different activities, 74 MBq vs. 148 MBq); (**) statistically significant difference (*p* < 0.05)—(comparison between treatments).

The radiation enhancement ratio (RER) was calculated and plotted in order to evaluate the radiosensitization effects after the combined treatment of Lu-177, as an internal irradiator, with AuNPs compared to plainLu-177 treatment. The results were analyzed and compared across the two Lu-177 activities (74 MBq and 148 MBq) and the two AuNP sizes (10 nm and 50 nm), as depicted in Figure 3.

Statistical analysis of the RER measurements showed significant differences for both comparisons made. A statistically significant difference was observed between the two Lu-177 activities (74 and 148 MBq), with a *p*-value of 0.01. Additionally, a significant difference was found between the two sizes of AuNPs (10 nm and 50 nm), with a *p*-value of 0.02.

### 3.3. Apoptosis Measurements

**Figure 4:**
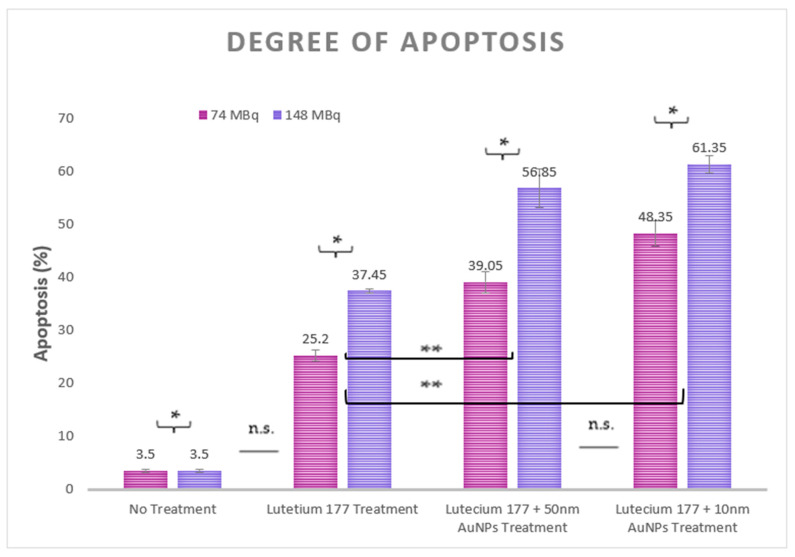
Apoptosis levels in HepG2 cells across different treatment conditions: control (untreated), Lu-177 alone, and Lu-177 combined with 10 nm or 50 nm AuNPs. Measurements were taken for two Lu-177 activities: 74 MBq (magenta) and 148 MBq (purple). (*) Statistically significant difference (*p* < 0.05)—(comparison between different activities, 74 MBq vs. 148 MBq), (**) statistically significant difference (*p* < 0.05)—(comparison between treatments), and (n.s.) non-significant difference (*p* > 0.05).

Apoptosis in HepG2 cells was quantified using flow cytometry to accurately assess and elucidate the extent of programmed cell death induced by ionizing radiation, both with and without the presence of AuNPs during Lu-177 therapy. Apoptosis was measured across several conditions: untreated control cells, cells treated with Lu-177 alone, and cells subjected to combined Lu-177 and AuNP therapy, with AuNP sizes of 10 nm and 50 nm, as depicted in Figure 4. These measurements were conducted for two different Lu-177 activities: 74 MBq and 148 MBq.

Statistical analysis of the apoptosis measurements revealed a significant difference between the two Lu-177 activities (74 and 148 MBq), with a *p*-value of 0.032. When comparing the different treatments, no statistically significant difference was observed between the no-treatment group and the Lu-177 treatment group (*p* = 0.06), nor between the combined treatment of Lu-177 with 10 nm AuNPs and the combined treatment of Lu-177 with 50 nm AuNPs (*p* = 0.1). However, a statistically significant difference was found between the Lu-177 treatment and the combined treatment groups of Lu-177 with 50 nm AuNPs (*p* = 0.05) as well as with 10 nm AuNPs (*p* = 0.005).

## 4. Discussion

### 4.1. Cell Survival Analysis

The results derived from the cell survival analysis demonstrate a substantial decrease in the survival fraction of HepG2 cells after the combined treatment of Lu-177 and AuNPs compared to that of Lu-177 alone (Figure 2). This reduction was observed across both activity levels (74 MBq and 148 MBq) and both sizes of AuNPs (10 nm and 50 nm). However, an evident superiority of the 10 nm AuNPs in increasing cell death was noted for both activities. This finding aligns with previous studies, which have shown that smaller AuNPs achieve higher intracellular deposition, particularly in cell organelles, such as lysosomes, mitochondria, and the nucleus [38,39,40]. The increased proximity of these smaller AuNPs to critical DNA-containing structures may enhance localized radiation effects, leading to more pronounced cytotoxicity and eventually greater cell death. This is supported by findings that smaller nanoparticles (around 10 nm) exhibit higher cellular uptake and accumulation in these organelles, potentially enhancing cytotoxic effects due to localized damage [38,39,40].

The presence of AuNPs enhance the cytotoxic effects of Lu-177, likely due to the increased local radiation dose generated by the interaction of gamma emissions with AuNPs. When irradiated with gamma photons, the high atomic number (Z) of gold increases the probability of the photoelectric effect, producing a cascade of secondary low-energy electrons, including Auger electrons. This damage manifests primarily as single-strand breaks (SSBs) and double-strand breaks (DSBs), which are critical for inducing cell death, particularly when repair mechanisms are overwhelmed [41,42,43]. Furthermore, AuNPs seem to increase reactive oxygen species (ROS) production, which adds to the oxidative stress on cancer cells, causing further indirect damage to DNA and other cellular structures [44,45]. However, in our case, it is crucial to recognize that, in addition to gamma emissions, short-range beta particles also play a role in the observed radiosensitization effects due to the use of AuNPS, and thus cannot be overlooked [46]. Given that the β-particles emitted by Lu-177 have a 2 mm range, they eventually present a cross-fire effect in their interactions with the surrounding environment, as the research team of Yook et al. observed in their experimental study [47]. Beta particles, with their higher mass and charge compared to photons, and due to their higher linear energy transfer (LET) than gamma rays, have a higher probability of direct interactions with AuNPs, leading to the production of secondary low-energy electrons that further contribute to direct DNA damage [48,49,50]. These secondary electrons can also ionize surrounding molecules and generate ROS, which induce oxidative stress and further enhance indirect cell killing [51,52]. Leung et al. showed that AuNPs significantly enhance dose effects in low-energy electron irradiations [53], which presents an analogy for the interaction with beta particles. The combination of these effects likely accounts for the enhanced cell death percentages observed in Figure 1, supporting the idea that AuNPs can effectively increase localized radiation damage if combined with therapeutic Lu-177.

The approach of plotting the survival fraction against activity levels attempts to align with nuclear medicine practices, where activity is the clinically relevant parameter, and to avoid the complexities of internal dosimetry calculations, especially in radiobiological cell survival models. This methodology facilitates a direct interpretation of therapeutic effects based on clinically applicable activity levels. Nevertheless, direct comparisons to other studies are limited due to the scarcity of the research exploring the use of AuNPs in combination with beta-emitting radionuclides, such as Lu-177, especially without incorporating functionalization methods. McKenna et al. conducted a similar investigation involving the combination of AuNPs with beta-emitters, like Yttrium-90, demonstrating an enhancement in local tumor dose deposition, which supports our findings. In contrast to these studies though, our research emphasizes leveraging the gamma emissions of Lu-177 to achieve additional therapeutic effects. This novel approach capitalizes on gamma radiation—typically considered non-therapeutic in nuclear medicine—to enhance treatment efficacy, offering a unique perspective in the field.

### 4.2. Radiation Enhancement Ratio (RER) Analysis

The radiation enhancement ratio (RER) calculations further confirmed the potential of AuNPs as effective radiosensitizers, especially when used in conjunction with Lu-177 therapy. Figure 3 demonstrates a significant increase in cell death in the presence of AuNPs, with more pronounced effects observed at higher activity levels of 148 MBq and with the smaller-sized 10 nm AuNPs. This finding further supports previous research indications that smaller nanoparticles, apart from higher cell death, also exhibit enhanced radiosensitization effects due to improved cellular uptake and localization [54,55,56]. The research team of Guo et al. observed that smaller AuNPs (around 10 nm) provided better radiosensitization in cancer cell lines, including HepG2, due to their higher cellular uptake and effective localization within critical organelles [57]. Chen et al. highlighted that the high atomic number of gold increases the probability of the photoelectric effect, generating secondary low-energy electrons that enhance localized DNA damage, which is crucial for effective radiosensitization [58]. Similarly, it has been reported that smaller AuNPs are more effective in enhancing radiation therapy, attributing this to their efficient cellular uptake and better targeting of critical cellular components [59,60].

Our study extends these findings to internal targeted therapy using Lu-177, where the AuNPs, particularly 10 nm in size, effectively amplify the radiation dose at a cellular level through local interactions with ionizing radiation, including the therapeutically non-useful gamma radiation of the radiopharmaceutical. Importantly, we also hypothesize the contribution of beta emissions, not only to the therapeutic result, but to the radiosensitrization effects. The high atomic number of gold (Z = 79) leads to a probability of interaction with charged particles, including beta electrons, due to the increased electron density around the nucleus [61]. This characteristic enhances the likelihood of energy absorption and subsequent ionization events, further augmenting the overall radiosensitization effects when Lu-177 is used in conjunction with AuNPs [62,63]. The increased RER observed at higher activity levels and with smaller nanoparticles underscores their enhanced ability to improve radiation therapy outcomes. This is consistent with previous research that demonstrated that smaller AuNPs offer greater radiosensitization in liver cancer cells, reinforcing the importance of nanoparticle size in optimizing therapeutic efficacy [64,65].

The choice of the RER as a metric, rather than the dose enhancement factor (DEF) or specific enhancement ratio (SER), reflects our focus on nuclear medicine clinically relevant parameters, such as activity levels, rather than purely absorbed doses. This approach is supported by Ricketts et al. who emphasized the significance of clinically relevant measurements in nanoparticle-enhanced radiotherapy studies [66]. Our results suggest that the addition of AuNPs can potentially allow for lower administered activities while achieving comparable or greater levels of tumor cell eradication, offering a substantial therapeutic advantage.

### 4.3. Apoptosis Measurements Analysis

The analysis of the apoptotic levels provided interesting insights into the underlying mechanisms of cell death under different Lu-177 treatment conditions. The highest levels of apoptosis were observed in cells treated with the combined Lu-177 and AuNP therapy, particularly with 10 nm AuNPs at 148 MBq. This finding is supported by recent research highlighting the enhanced radiosensitization effects of smaller AuNPs in HepG2 cells. Further studies demonstrated that smaller gold nanoparticles significantly increased apoptosis in HepG2 cells when used in conjunction with radiation therapy, attributing this effect to improved cellular uptake and localized dose enhancement [67,68]. Similarly, Zhao et al. found that AuNPs were more effective in inducing apoptosis through augmented radiation damage and oxidative stress [69]. Research teams also observed AuNPs’ size dependency regarding levels of apoptosis in cancer cell lines, corroborating the importance of nanoparticle size in enhancing therapeutic efficacy [70]. Additionally, the research reports that gold nanoparticles induce programmed cell death through oxidative stress-mediated apoptosis, further supporting the role of AuNPs in enhancing radiation-induced apoptosis [71,72,73,74].

Measuring apoptosis is crucial for verifying that the observed cell death and radioenhancement effects are indeed a result of ionizing radiation and of the secondary effects of AuNPs. Apoptosis, or programmed cell death, is a direct indicator of cellular damage induced by ionizing radiation. The enhanced apoptosis observed in our study corroborates the hypothesis that the localized dose amplification caused by AuNPs leads to increased DNA damage and oxidative stress, which are key mechanisms in radiation-induced cell death. By quantifying apoptosis, we can confirm that the increased cell death and radiosensitization effects are attributable to the combination of Lu-177’s gamma and beta emissions and AuNPs, rather than other factors. This provides a clear link between ionizing radiation and the secondary biological effects mediated by AuNPs, validating their role as effective radiosensitizers in enhancing therapeutic outcomes.

### 4.4. Limitations and Future Perspectives

While this study presents promising findings regarding the enhanced efficacy of Lu-177 radiopharmaceutical therapy in combination with AuNPs, a number of limitations must be acknowledged toward the direction of future research optimization.

Lu-177 emits both gamma and beta radiation, and both can play a role in the observed AuNPs radioenhancement and subsequent cell death. To accurately quantify and discern the relative contributions of gamma and beta radiation in this radiosensitization effect, further analysis of the underlying mechanisms of radiation-induced cellular damage is necessary, including their respective roles in contributing to both direct and indirect DNA breaks, as well as the disruption of cellular homeostasis through oxidative stress and ROS production. Future research should incorporate techniques, such as ROS measurement, to elucidate how gamma and beta rays interact with AuNPs when combined with Lu-177. More specifically, insights regarding the fraction of radiation absorbed and converted into excitation or ionization events, meaning the generation of ROS, which can be influenced by factors like AuNP size, incubation time, or concentration are valuable. Additionally, it is beneficial for future studies to focus on markers of lipid peroxidation caused by oxidative radicals, providing a more targeted measure of oxidative damage. This would enhance our understanding of the oxidative stress induced by the combined treatment of Lu-177 and AuNPs. Beyond oxidative stress, investigating the acute effects of Lu-177 and AuNPs on cellular energy metabolism, particularly the uncoupling of oxidative phosphorylation, is a promising avenue for future research [75]. Since energy depletion is a critical factor in triggering apoptosis, understanding this mechanism enhances our current knowledge of the cytotoxic pathways activated by Lu-177 and AuNPs [76]. Taken together, these additional approaches would offer a more comprehensive view of the cellular responses to our treatment strategy.

An important aspect of understanding the full therapeutic potential of the combined treatment with Lu-177 would also be the evaluation of their effects on the cell cycle. Previous research has indicated that AuNPs alone can induce cell cycle arrest, particularly in the G2/M phase, thereby making cells more susceptible to radiation-induced damage [77]. However, investigations specifically examining the combined impact of AuNPs and Lu-177 on the cell cycle are still limited. Understanding whether the nanoparticles alone or in combination with Lu-177 affect cell cycle progression could provide valuable insights into the mechanisms of radiosensitization and cell death.

Additionally, assessing the effects of the combination of Lu-177 and AuNPs on a normal cell line with a relevant cellular morphology to hepatocellular carcinoma would be highly beneficial. Previous studies have shown that Lu-177 can exhibit varying degrees of cytotoxicity in normal cells, influenced by factors such as radiation dose, exposure duration, and cell type. Thus, understanding the impact of Lu-177 on healthy cells, in conjunction with AuNPs, is critical for establishing a therapeutic window where cancer cells are effectively targeted while minimizing damage to normal tissues, ultimately enhancing patient safety and treatment outcomes. In a prior in vitro study, we investigated the effects of AuNPs on the HCK1T human cervical keratinocyte cell line [29]. Our findings reveal that, while cancer cells exhibit increased radiosensitivity and higher death rates, normal HCK1T cells demonstrate enhanced DNA repair capabilities [29]. Therefore, we anticipate minimal effects of AuNPs on healthy cells, aside from potential toxicity due to deposition. However, evaluating their impact on hepatic healthy cell lines is essential, as both cell type and AuNP characteristics can significantly influence outcomes. Our results indicate that AuNPs substantially decrease the survival rates in cancer cells while sparing normal cells, supporting the notion that AuNPs preferentially accumulate in tumors via the enhanced permeability and retention (EPR) effect. Additionally, the research team of Georgiou et al. demonstrated that the combination of Lu-177 and AuNPs, when administered via convection-enhanced delivery (CED), had a minimal impact on healthy tissues in a laboratory mouse model [78]. High tumor retention of Lu-177-AuNPs was observed, with little redistribution to the surrounding normal brain tissue or other organs [78]. No signs of toxicity were noted in normal tissues, and no visible damage was detected on MRI scans or histological staining [78]. These results indicate that Lu-177-AuNPs selectively target tumor cells while sparing healthy tissues from significant radiation exposure or damage. Nevertheless, further research on this subject remains necessary.

## 5. Conclusions

This study aimed to explore the effects of Lu-177 radiopharmaceutical with AuNPs on the HepG2 hepatic cancer cell line. It offers a novel perspective compared to the existing research that primarily focuses on functionalizing AuNPs with Lutetium. Instead of directly conjugating Lu-177 to the nanoparticles, our approach utilized Lu-177 as an internal irradiator, leveraging its beta and gamma emissions for localized radioenhancement.

The findings demonstrate that this combined treatment strategy effectively utilizes beta but also gamma emissions—typically considered therapeutically redundant in the field of nuclear medicine therapy. By amplifying the radiation activity, specifically within the tumor microenvironment, the study confirms that the combination of Lu-177 and AuNPs significantly increases cell death and apoptosis. This approach provides a unique method to improve therapeutic efficacy without the need for complex nanoparticle modifications.

Our results highlight the potential of this novel strategy to not only utilize Lu-177 beta radiation, but also transform otherwise non-therapeutic gamma radiation into a targeted therapeutic advantage. The enhanced radioenhancement observed, particularly with smaller-sized AuNPs (10 nm compared to 50 nm), underscores the importance of size-dependent effects and localized cell death amplification. However, further research is essential to elucidate the individual contributions of gamma and beta radiation to the observed radiosensitization effects, as well as to comprehensively evaluate their impacts on cell cycle dynamics. Additionally, it is crucial to assess the effects on normal cell lines, particularly those relevant to hepatocellular carcinoma. A deeper understanding of these interactions will facilitate the optimization of treatment protocols, enhance therapeutic efficacy, and ultimately improve patient safety. Eventually, this research aims to contribute valuable insights into optimizing neuroendocrine cancer therapies and lays the groundwork for future studies to translate these findings into clinical applications. By advancing toward personalized therapy, we focus on localized and enhanced cell death through increased apoptosis and sparing healthy tissue with the aid of AuNPs. The study highlights a novel approach to radionuclide therapies, demonstrating the promising potential of utilizing gamma emissions in radiopharmaceuticals to activate AuNP-mediated radioenhancement mechanisms, enabling a more targeted and effective cancer treatment.

## Data Availability

Data are available from the corresponding authors upon reasonable request.
